# Non-Invasive Bedside Assessment of Central Venous Pressure: Scanning into the Future

**DOI:** 10.1371/journal.pone.0109215

**Published:** 2014-10-03

**Authors:** Jacques Rizkallah, Megan Jack, Mahwash Saeed, Leigh Anne Shafer, Minh Vo, James Tam

**Affiliations:** 1 Department of Medicine, section of Cardiology, University of Manitoba, Winnipeg, Manitoba, Canada; 2 University of Manitoba Medical School, University of Manitoba, Winnipeg, Manitoba, Canada; 3 Department of Medicine, Health Sciences Center, University of Manitoba, Winnipeg, Manitoba, Canada; Medical University of Graz, Austria

## Abstract

**Background:**

Noninvasive evaluation of central venous pressure (CVP) can be achieved by assessing the Jugular Venous Pressure (JVP), Peripheral Venous Collapse (PVC), and ultrasound visualization of the inferior vena cava. The relative accuracy of these techniques compared to one another and their application by trainees of varying experience remains uncertain. We compare the application and utility of the JVP, PVC, and handheld Mini Echo amongst trainees of varying experience including a medical student, internal medicine resident, and cardiology fellow. We also introduce and validate a new physical exam technique to assess central venous pressures, the Anthem sign.

**Methods:**

Patients presenting for their regularly scheduled echocardiograms at the hospital echo department had clinical evaluations of their CVP using these non-invasive bedside techniques. The examiners were blinded to the echo results, each other's assessments, and patient history; their CVP estimates were compared to the gold standard level 3 echo-cardiographer's estimates at the completion of the study.

**Results:**

325 patients combined were examined (mean age 65, s.d. 16 years). When compared to the gold standard of central venous pressure by a level 3 echocardiographer, the JVP was the most sensitive at 86%, improving with clinical experience (p<0.01). The classic PVC technique and Anthem sign had better specificity compared to the JVP. Mini Echo estimates were comparable to physical exam assessments.

**Conclusions:**

JVP evaluation is the most sensitive physical examination technique in CVP assessments. The PVC techniques along with the newly described Anthem sign may be of value for the early learner who still has not mastered the art of JVP assessment and in obese patients in whom JVP evaluation is problematic. Mini Echo estimates of CVPs are comparable to physical examination by trained clinicians and require less instruction. The use of Mini Echo in medical training should be further evaluated and encouraged.

## Introduction

Noninvasive evaluation of central venous pressure (CVP) is a component of the physical examination that can be very valuable in patient care, especially in the assessment of volume status. CVP estimates can be achieved by assessing the Jugular Venous Pressure (JVP), Peripheral Venous Collapse (PVC), and ultrasound visualization of the inferior vena cava (IVC).

**Figure 1:**
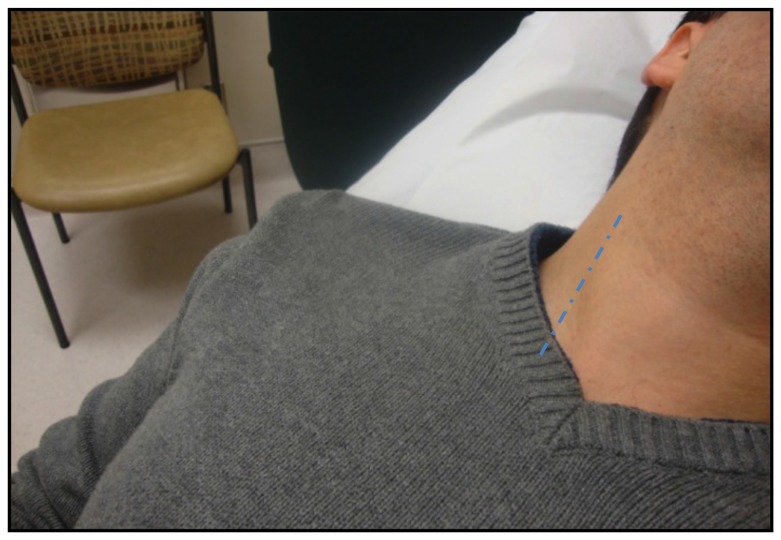
Assessment of Jugular Venous Pressure. The doted line displays the course of the internal jugular vein between the 2 heads of the sternocleidomastoid muscle.

**Figure 2:**
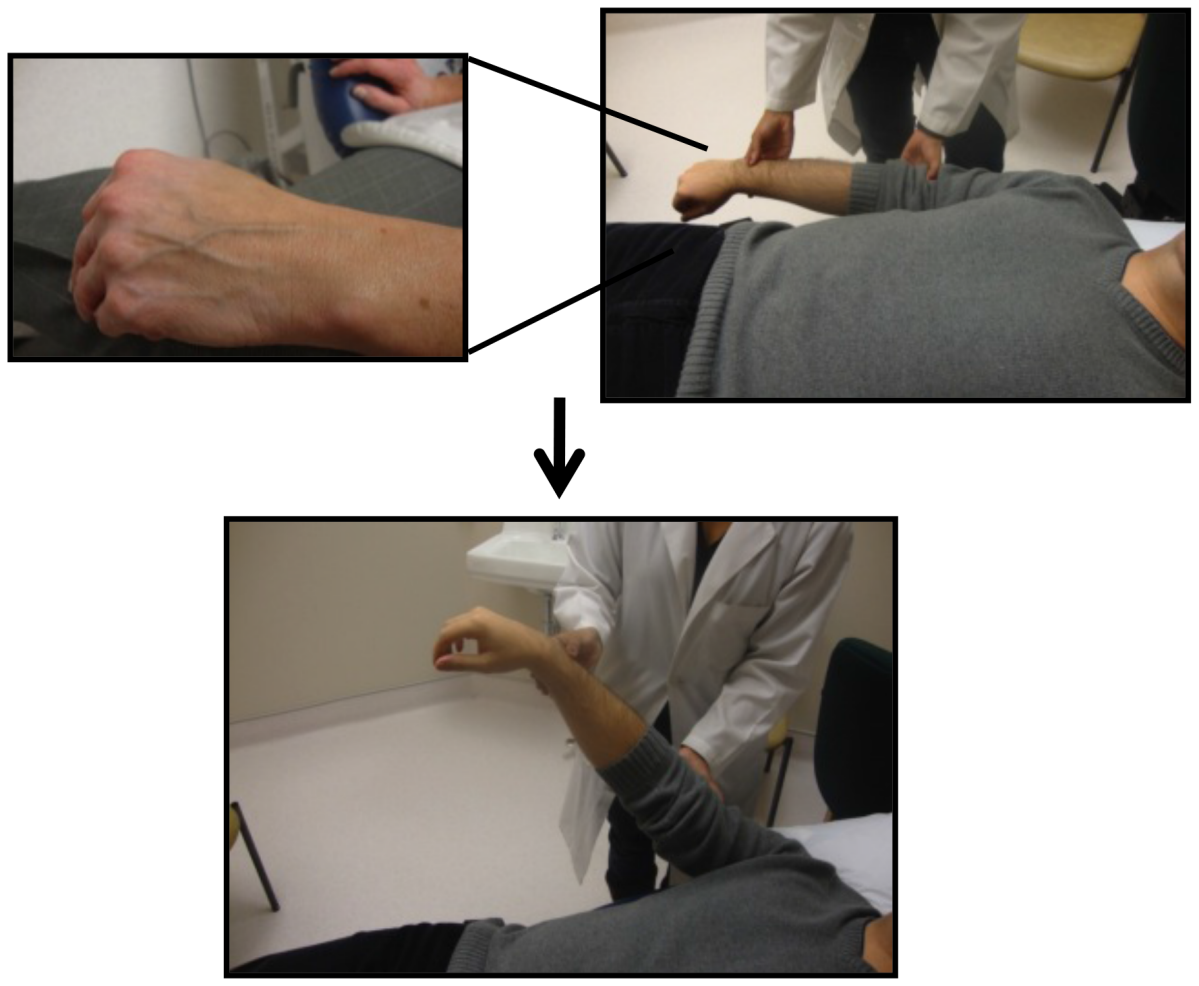
Assessment of Peripheral Venous Collapse; the classical peripheral venous collapse technique.

Bedside evaluation of CVP dates back to the 1920s following Starling's cardiac hemodynamic experiments linking it to cardiac outputMcGee [1]. The height of the JVP (Figure 1) provided a useful estimate of the CVP, which in turn gives a rough correlate of patient's volume status [2]. The CVP is considered elevated when the height of the internal or external JVP is >3 cm of vertical distance above the sternal angle [1]–[4]. The use of peripheral vein collapse on the dorsum of the hand or antecubital fossae was described as an alternate estimate of CVP [5]–[7]. Using the sternal angle as a reference point, the arm is slowly elevated passively from a dependent position and if PVC occurs above the sternal angle, CVP is considered elevated [5] (Figure 2).

JVP evaluation can be very challenging due to various factors including obesity, anomalous venous anatomy, connective tissue diseases, and venous scarring from catheter insertion [8]. In such cases, the PVC technique could potentially be an alternative non-invasive measure of CVP. To our knowledge, the PVC technique has never been validated as a physical exam tool, especially in relation to other non-invasive methods [9]. The relative accuracy of these techniques and their application by trainees remains uncertain.

**Figure 3:**
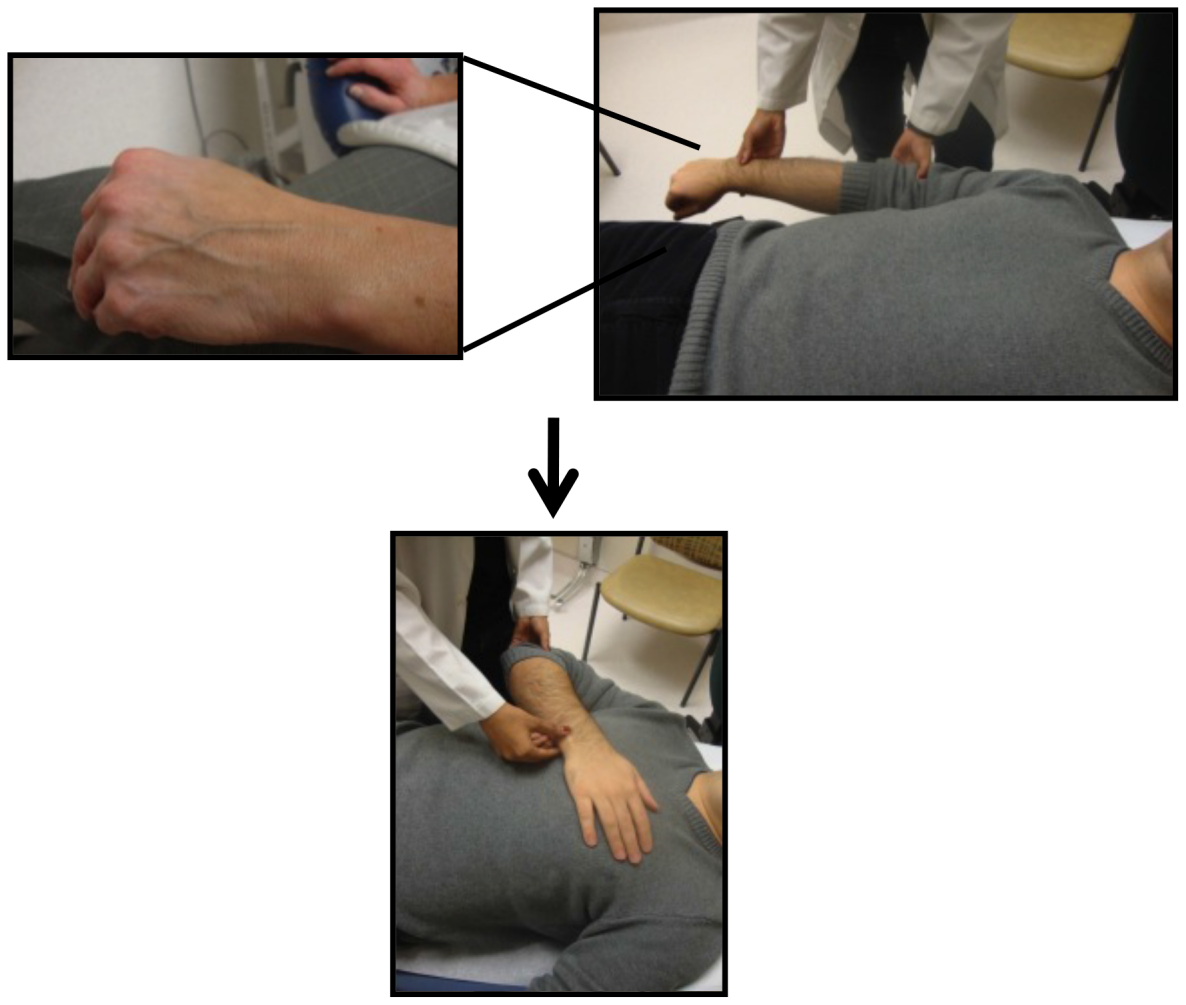
Assessment of Peripheral Venous Collapse; the Anthem sign.

In this study we evaluate the application and utility of the JVP, PVC, and handheld Mini Echo as non-invasive CVP clinical predictive tools amongst trainees of varying experience. We also introduce and validate a new physical exam technique to assess central venous pressures, the Anthem sign (Figure 3).

## Methods

This study was conducted at St Boniface Hospital's Echo department in Winnipeg, Manitoba, with formal ethics approval from the University of Manitoba. A cohort of in- and out-patients presenting for their regularly scheduled echocardiograms provided consent for clinical evaluation of CVP using the non-invasive bedside techniques; these included the JVP, PVC techniques, and the handheld mini Echo. We compared the application and utility of these techniques amongst three trainees of varying experience; a 2^nd^ year medical student with limited clinical experience, a 2^nd^ year medical resident with 3 years of clinical experience, and a 2^nd^ year cardiology fellow with 6 years of clinical experience. Patients were excluded if they had intravenous catheters in the right-sided veins and/or were not able to give informed consent.

All formal echocardiograms were completed by a trained sonographer and interpreted by a level 3 echo-cardiographer who established the reference CVP based on the evaluation of the IVC's caliber and response to respiration as recommended in the 2010 American Society of Echo guidelines [10]; IVC diameter ≤2.1 cm collapsing >50% with sniff suggests normal right atrial pressure of 3 mmHg with a range of 0 to 5 mmHg [10]; IVC diameter >2.1 cm collapsing <50% with sniff suggests high right atrial pressure of 15 mmHg with a range of 10–20 mmHg [10]. The estimate of CVP by the research echocardiographer nearly identically matched the CVP provided within the clinical interpreter of the clinical echo report. The examiners were blinded to the echo results, each other's assessments, and patient history; their CVP estimates were compared to the gold standard level 3 echo-cardiographer's estimates at the completion of the study.

The individual in this manuscript images has given written informed consent (as outlined in PLOS consent form) to publish these case details.

### Ethics Statement

This research project conforms with the World Association's Declaration of Helsinki and has been approved by the University of Manitoba Bannatyne Campus Research Ethics Board. Written patient informed consents were also obtained prior to enrolment in the study.

The individual in this manuscript images has given written informed consent (as outlined in PLOS consent form) to publish these case details.

### Description of Non-Invasive Bedside Assessment Techniques

#### 1. Jugular Venous Pressure evaluation:

CVP estimates are obtained by determining the height of the internal jugular venous waveforms relative to the sternal angle (Figure 1). CVPs are considered elevated when the height of the venous column is >3 cm above the sternal angle [11]. The right internal jugular vein was initially evaluated since it communicates with the right atrial in a relatively straight course. If the venous wave-forms of the right internal jugular vein were not well visualized, the left internal jugular vein was evaluated. For the sake of consistency within this study, we did not examine the external jugular vein although prior studies have demonstrated its reliability in estimating CVP in some studies [12]–[14].

#### 2. Peripheral Venous Collapse techniques:

In the supine position with a 30 degree elevation in the head of the bed, the patient's arms are rested on the side of the body and the dorsum of the hand is inspected for superficial veins (Figure 2). If the veins are not visible, this technique cannot be applied and if visible but collapsed already the CVP is likely low or normal. If the veins are distended the arm is passively elevated and the level of PVC relative to the sternal angle is noted. If the veins remain distended above the sternal angle the CVP is likely elevated. Because the observation point of PVC on the dorsum of the hand and the reference point at the sternal angle are quite far apart, the precise determination of the level of PVC is subject to error. In an attempt to overcome this limitation, we modified the classic PVC technique and introduced and validated a new physical exam method called the Anthem or Rizkallah sign (Figure 3). This new technique begins in a similar fashion as the classic PVC method. However, when the arm is ready to be passively elevated, it is simply placed directly over the sternum. In this approach, the PVC observation point on the dorsum of the hand and the reference point at the sternum are in close proximity and if the veins remain distended while rested on top of the sternum the CVP is thought to be elevated. Since in this position the patient appears as an individual standing in attention for a national anthem, this method was named the Anthem sign.

#### 3. Bedside Mini-Echo:

**Figure 4:**
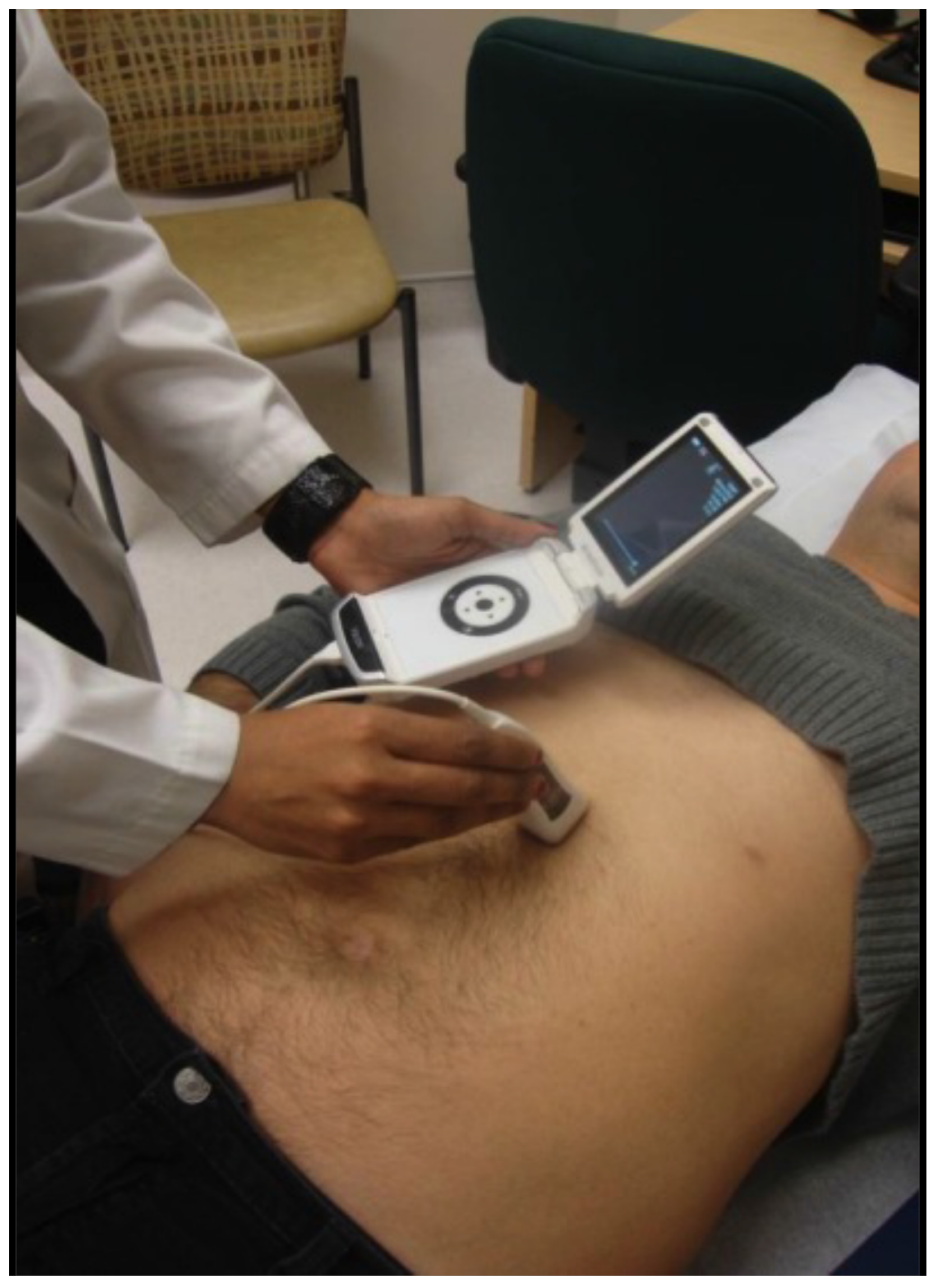
Hand-held bedside ultrasound device (Mini Echo).

A handheld ultrasound device (Figure 4) was utilized to assess the IVC to estimate CVPs as outlined by the American Society of Echo 2010 guidelines [10].

### Trainee Education

Prior to patient recruitment for the study, the medical student received focused training on the application of the JVP and PVC techniques by examining 44 patients over a period of 1 week, totaling an estimated teaching time of 10 hours. The internal medicine resident only received very brief instruction on the application of the physical exam techniques based on a patient assessment. Finally, the cardiology fellow did not require tutelage on the application of the bedside physical exam techniques. The Mini Echo was only utilized by the medical student following 10 hours of Echo training. The research took place during the summer project between second and third year medical school for the medical student and during a dedicated 4 week research rotation for the medical resident and the cardiology resident.

Forty-two patients were examined by both the medical student and resident independently to assess inter-observer agreement.

### Statistical Analysis

The data was analyzed using ordinary linear and logistic regressions for comparison with the gold standard of echocardiographic assessment, concordance correlations for continuous variables, sensitivity and specificity analysis for comparison of categorical variables with the gold standard, and Fisher Exact tests to compare differences in sensitivity and specificity across different groups. Overall agreement was computed as the proportion of correct evaluations, both negative and positive, when compared with the gold standard estimates of CVP by the level 3 echocardiographer.

## Results

### Overall patient characteristics (Table 1)

In total, 325 patients combined were examined: 217 by the medical student, 58 by the medical resident, 49 by the cardiology fellow, and 43 evaluated using Mini-Echo assessments. The average age was 65 (s.d.16) years with a mean BMI of 28 kg/m^2^ (s.d.6), and 52% were males (Table 1). Seventy-eight percent (78%) had abnormal echocardiograms, 26% LV systolic dysfunction with 5% being severe, 17% RV dysfunction with 2% being severe, and 3% with more than mild tricuspid regurgitation. CVP assessments by the level 3 echo-cardiographer were 6 (s.d.3) mmHg (normal  = 3–5 mmHg). 30% of the patients had elevated CVPs based on the gold standard IVC assessment by the level 3 echo-cardiographer with the exception for the 43 patients scanned by the Mini-Echo for which the prevalence was close to 47%.

**Table 1:** Overall patient characteristics.

Demographics and 2D-Echo parameters	Total Patient Population (n = 325)	Medical Student (n = 217)	Medical Resident (n = 58)	Cardiology Fellow (n = 49)	Medical Student Mini-Echo (n = 43)
Mean Age (years)	65±16	65±17	62±16	64±16	65±15
Males	52%	54%	53%	59%	35%
Mean BMI (kg/m^2^)	28±6	28±6	28±5	27±9	28±5
Mean Height of SA to mid-axillary line (cm)	11±3	10±2	14±3	13±2	n/a
Abnormal ECHO	252 (78%)	170 (78%)	47 (81%)	40 (82%)	26 (60%)
Normal LV function	239 (74%)	162 (75%)	40 (69%)	34 (69%)	34 (79%)
Mild LV dysfunction	45 (14%)	29 (13%)	8 (14%)	8 (16%)	4 (9%)
Moderate LV dysfunction	23 (7%)	15 (7%)	4 (7%)	3 (6%)	4 (9%)
Severe LV dysfunction	14 (4%)	9 (4%)	4 (7%)	4 (8%)	1 (2%)
LV function not available	4 (1%)	2 (1%)	2 (3%)	0 (0%)	0 (0%)
Normal RV function	270 (83%)	189 (87%)	47 (81%)	33 (67%)	39 (91%)
Mild RV dysfunction	25 (8%)	13 (6%)	2 (3%)	11 (22%)	1 (2%)
Moderate RV dysfunction	14 (4%)	11 (5%)	3 (5%)	4 (8%)	1 (2%)
Severe RV dysfunction	8 (2%)	2 (1%)	2 (3%)	0 (0%)	1 (2%)
RV function not available	8 (2%)	2 (1%)	4 (7%)	1 (2%)	1 (2%)
Normal/Mild TR	311 (96%)	211 (97%)	56 (97%)	43 (88%)	43 (100%)
Moderate TR	6 (2%)	2 (1%)	0 (0%)	4 (8%)	0 (0%)
Severe TR	4 (1%)	2 (1%)	1 (2%)	2 (4%)	0 (0%)
TR data not available	4 (1%)	2 (1%)	1 (2%)	0 (0%)	0 (0%)
Mean CVP from IVC ECHO assessment (mmHg)	6±3	6±2	6±2	6±3	6±2
% Normal/Low JVP	76%	84%	55%	45%	n/a
%Elevated JVP	18%	8%	33%	55%	n/a
% Not Available JVP	7%	8%	12%	0%	n/a

Examiner 1 = 2^nd^ year medical student.

Examiner 2 = 2^nd^ year internal medicine resident.

Examiner 3 = 2^nd^ year cardiology fellow.

BMI =  Body Mass Index, CVP =  Central venous pressure, IVC =  Inferior Vena Cava, JVP =  Jugular Venous Pressure, LV =  Left ventricle, n/a =  not applicable, RV =  Right ventricle, TR =  Tricuspid regurgitation.

### Application of non-invasive bedside clinical exam techniques

When examining the JVP, the medical student, medical resident, and cardiology fellow concluded that the CVP was elevated in 8%, 33%, and 55% of their respective patients (Table 1). Of all the physical examination techniques, the JVP was the most sensitive for detecting elevated CVPs with sensitivities improving with clinical experience from 13%, 53%, to 86% when evaluated by the medical student, resident, and cardiology fellow, respectively (p<0.01) (Table 2). This rising trend in sensitivity with clinical experience was partly, but not completely, offset by a declining trend in specificity (Table 2).

**Table 2:** Sensitivity and specificity of the physical exam techniques in the general patient population.

Evaluation of non-invasive physical exam techniques	Medical Student (n = 217)	Medical Resident (n = 58)	Cardiology Fellow (n = 49)	Medical Student Mini-Echo (n = 43)
JVP agreement with gold standard	72%	65%	65%	n/a
PVC agreement with gold standard	68%	67%	66%	n/a
Anthem sign agreement with gold standard	69%	65%	72%	n/a
Mini-Echo agreement with gold standard	n/a	n/a	n/a	72%
JVP sensitivity	13%	53%	86%	n/a
PVC sensitivity	15%	8%	50%	n/a
Anthem sign sensitivity	21%	15%	38%	n/a
Mini-Echo sensitivity	n/a	n/a	n/a	100%
JVP specificity	93%	71%	57%	n/a
PVC specificity	89%	91%	71%	n/a
Anthem sign specificity	88%	85%	85%	n/a
Mini-Echo specificity	n/a	n/a	n/a	66%
JVP PPV	39%	47%	44%	n/a
PVC PPV	35%	25%	38%	n/a
Anthem sign PPV	39%	29%	50%	n/a
Mini-Echo PPV	n/a	n/a	n/a	40%
JVP NPV	75%	75%	91%	n/a
PVC NPV	73%	71%	80%	n/a
Anthem sign NPV	74%	72%	78%	n/a
Mini-Echo NPV	n/a	n/a	n/a	100%

Examiner 1 = 2^nd^ year medical student.

Examiner 2 = 2^nd^ year internal medicine resident.

Examiner 3 = 2^nd^ year cardiology fellow.

JVP =  Jugular Venous Pressure, n/a =  not applicable, NPV =  Negative Predictive Value, PPV =  Positive Predictive Value, PVC =  Classic Peripheral Venous Collapse technique.

When applied by the examiner with the least clinical experience, the PVC techniques, with the Anthem sign in particular, had greater sensitivity than the JVP at 21% vs 13% (Table 2). Similar trends were observed in the evaluation of obese patients (Table 3). After only brief echo training, the Mini-Echo assessments by the medical student were the most sensitive, at 100%.

**Table 3:** Sensitivity and specificity of the physical exam techniques in the obese patients (BMI >30 kg/m^2^).

Evaluation of non-invasive physical exam techniques	Medical Student (n = 71)	Medical Resident (n = 17)	Cardiology Fellow (n = 10)	Medical Student Mini-Echo (n = 15)
JVP agreement with gold standard	59%	62%	30%	n/a
PVC agreement with gold standard	57%	64%	40%	n/a
Anthem sign agreement with gold standard	61%	64%	70%	n/a
Mini-Echo agreement with gold standard	n/a	n/a	n/a	67%
JVP sensitivity	5%	100%	100%	n/a
PVC sensitivity	0%	0%	100%	n/a
Anthem sign sensitivity	15%	0%	50%	n/a
Mini-Echo sensitivity	n/a	n/a	n/a	100%
JVP specificity	84%	44%	13%	n/a
PVC specificity	85%	82%	33%	n/a
Anthem sign specificity	83%	82%	75%	n/a
Mini-Echo specificity	n/a	n/a	n/a	58%
JVP PPV	13%	44%	22%	n/a
PVC PPV	0%	0%	29%	n/a
Anthem sign PPV	30%	0%	33%	n/a
Mini-Echo PPV	n/a	n/a	n/a	38%
JVP NPV	65%	100%	100%	n/a
PVC NPV	63%	75%	100%	n/a
Anthem sign NPV	67%	75%	86%	n/a
Mini-Echo NPV	n/a	n/a	n/a	100%

Examiner 1 = 2^nd^ year medical student.

Examiner 2 = 2^nd^ year internal medicine resident.

Examiner 3 = 2^nd^ year cardiology fellow.

JVP =  Jugular Venous Pressure, n/a =  not applicable, NPV =  Negative Predictive Value, PPV =  Positive Predictive Value, PVC =  Classic Peripheral Venous Collapse technique.

The Anthem sign had the most consistency in its sensitivity and specificity when applied by all 3 examiners. When applied by the cardiology fellow, the Anthem sign had higher specificity than the JVP (85% vs 57% respectively) (Table 2). Among all three trainees, the classic PVC technique and Anthem sign had better specificity compared to the JVP, especially among obese patients.

The positive predictive values for all 3 non-invasive clinical exam techniques when utilized by the most skilled learner, the cardiology fellow, were fairly similar but with a trend towards better performance for the Anthem sign both in the general and obese patient population; the JVP had the highest negative predictive values at 91% when compared to the other PVC techniques (Tables 2 and 3).

In terms of inter-observer agreement, the patterns of sensitivities and specificities for bedside clinical exam techniques were similar in the 42 co-examined patients, as compared to the complete sub-group of patients examined by the medical student and resident (Table 4).

**Table 4:** Sensitivity and specificity of the physical exam techniques in the 42 co-examined patients.

Evaluation of non-invasive physical exam techniques	Medical Student (n = 42)	Medical Resident (n = 42)
JVP sensitivity	0%	58%
PVC sensitivity	14%	12.5%
Anthem sign sensitivity	14%	25%
JVP specificity	87%	68%
PVC specificity	71%	68%
Anthem sign specificity	68%	71%

Examiner 1 = 2^nd^ year medical student.

Examiner 2 = 2^nd^ year internal medicine resident.

CVP =  Central venous pressure, JVP =  Jugular Venous Pressure, PVC =  Classic Peripheral Venous Collapse technique.

## Discussion

In this study we assessed the application and utility of various non-invasive bedside clinical exam techniques to estimate central venous pressures amongst trainees of varying experience; these included the JVP, PVC techniques, and IVC assessments by handheld Mini Echo. We also validated the classically described PVC technique and introduced and validated a new physical exam technique to assess central venous pressures, the Anthem sign.

JVP assessment was the most reliable amongst the 3 physical exam techniques, with improving sensitivity with clinical experience. The internal jugular veins, right sided in particular, communicate with the right atrium in a relatively straight course making JVP assessment a better physical exam tool compared with the PVC techniques; JVP assessment avoids the influence of various bends and obstructions in the course of the arm veins along with the effect of a greater number of valves on CVP transmission. The JVP however can be a difficult skill to master due to challenges in differentiating venous waveforms from carotid pulsations; the various maneuvers that help identify the jugular venous waveforms take time and practice to master as demonstrated by the results of the 3 trainees of varying experience (Table 2). On the other hand, amongst our 3 trainees the application of the PVC techniques was an easier skill to acquire by the early learner; this was reflected by the better performance of these techniques relative to the JVP evaluation at this early stage in training. Although the application of all 3 physical exam techniques by the medical student yielded low sensitivities the above observations suggest that the PVC techniques can be good adjunctive tools in the evaluation of CVPs until the art of assessing the JVP has been mastered; they also have the potential to be good adjunctive tools for the experienced examiner as evidenced by the relatively higher positive predictive values as compared to the JVP application in the study population as a whole (Table 2) and in the obese patient population (Table 3); this is of particular value in the evaluation of obese patients where the jugular venous waveforms are difficult to visualize due to adiposity. The application of the PVC techniques requires some tutelage as suggested by better CVP estimates of examiner 1 who received training evaluations on 42 volunteers compared to the examiner 2 who only applied the techniques on 1 training volunteer prior to study enrollment. In addition, it is a skill that is acquired faster than the mastery of the JVP evaluation.

The application of the Anthem sign seemed to yield the most consistent estimates of CVPs amongst all 3 examiners when compared to the classic PVC technique and the JVP assessment as demonstrated by the relatively similar sensitivities (Table 2). It is a technique that is very easy to apply with the only challenge being determining the suitability of the hand veins for evaluation (Figure 3).

The use of the Mini-Echo by the medical student to estimate CVPs after brief training sessions was comparable and even superior to the most sensitive bedside technique even when applied by the most skilled examiner; the sensitivity of estimating CVPs based on the JVP was 86% for the cardiology fellow as compared to 100% by the student's Mini-Echo. The utilization of handheld echocardiography does not require intensive training as demonstrated in our study and also in other published reports [15], [16]. For instance, in one study 3 medicine residents were able to use the handheld echo device as a general screening tool for cardiac disease after 20 hours of didactic instruction and practice on 20 volunteers [15]. In another, 13 medicine residents learned to assess LV systolic function using a handheld ultrasound device after only 1 hour of training [16]. The use of a handheld ultrasound device should not outweigh the importance of a formal complete echocardiographic evaluation by trained sonographers. Nonetheless, it can be a very helpful adjunct for a complete physical exam and its introduction in trainee formation should be further evaluated.

Three examiners were involved in the study, one in each experience group, which may limit the application of our findings to all learners in the respective experience groups. However, having many examiners in each experience group assess the same patients would have been disruptive to the daily busy workload of the echo lab and made patient recruitment more difficult. The observed improvement in JVP assessment with clinical experience by the more senior examiners suggests acceptable representation by our examiners of their respective experience groups.

Ideally all 3 examiners should be examining all the recruited patients independently but this would also inconvenience patients and make recruitment very difficult. To help address this issue in study design, forty-two patients were examined by both the medical student and resident independently to assess inter-observer agreement. The patterns of sensitivities and specificities for bedside clinical exam techniques were similar in the 42 co-examined patients as compared to the complete sub-group of patients as described in Table 4. These observations make a significant patient-related bias less likely.

An ideal gold standard for assessments of CVP would be invasive measurements using a manometered tip catheters; however, patient recruitment would be limited to critical care settings thus limiting generalizability of findings especially away from patients for whom application of non-invasive bedside clinical exam techniques are most useful. In addition, subjecting clinically stable patients to invasive CVP measurements would be impractical and unethical considering the potential risks of catheter insertion. Ultrasound guided CVP assessment as recommended by the American Society of Echo was thus selected as a good alternative gold standard for this study [10]. The use of echo assessment of the IVC to estimate central venous pressures is a well-accepted and reliable non-invasive technique that has been favorably validated relative to invasive hemodynamic measurements by numerous past and recent studies [9], [17]–[34]. In a low risk cohort of patients, as the present one, it is the only ethically acceptable gold standard rather than subjecting these patients to the risks of invasive catheterization and testing.

In the appropriate clinical setting however, the use of invasive measurements of CVP has its merit. This is particularly the case in certain critical care cases or intra-operatively where rapid and frequent assessments of CVP are required to tailor therapies and where the application of non-invasive techniques may not be very practical. In these cases, the risks associated with invasive measurements of CVP are minimal compared to the potential benefits of rapid hemodynamic evaluation required for emergent care. When an invasive CVP evaluation method is chosen it should be discontinued as soon as possible to minimize the risk of potential complications. Depending on the type of central venous catheter utilized and method of insertion, complications associated with invasive CVP measurements will primarily center on catheter insertion and could include bleeding near the puncture site, infection, vessel injury including arterio-venous fistula formation, local venous or arterial thrombosis along with the associated embolic risk including stroke, foreign body embolus from the catheter insertion kit, pneumothorax, hemothorax, along with atrial or ventricular arrhythmias amongst others [35]–[43]. Due to such potential risks associated with invasive CVP measurements, a non-invasive gold standard was deemed more appropriate for our low risk patient cohort.

In terms of the application of most physical exam techniques, trends in sensitivity and specificity do not always mirror one another. A trend in one direction for sensitivity would very likely be coupled with a trend in the other direction for specificity; this observed heterogeneity in sensitivity and specificity of the various techniques suggests their potential complementary role when used as adjuncts to one another.

When applying a physical examination technique at the bedside, it is important to be aware of its practical limitations prior to interpreting and integrating its results clinically. For instance, the evaluation of the JVP technique to estimate a heart failure patient's volume status may be misleading in the setting of underlying severe tricuspid regurgitation. In this particular scenario, one has to carefully differentiate the venous wave-forms of the jugular vein as the “a” wave produced by atrial contraction will be a better reflection of central venous pressure compared the “v” wave which is reflective of tricuspid regurgitation; such a distinction may be hampered in patients with atrial fibrillation in whom an “a” wave is seldom observed [8], [44]. Another important consideration in the evaluation of the JVP technique is the at times compromised integrity of the internal jugular vein valve which may lead to misleading waveform interpretation; this is often the case in patients with a central venous catheter insertion within the vein itself or in the setting of degenerative jugular vein valvular insufficiency [8], [45]–[48]. Similarly, when evaluating the peripheral venous collapse techniques to estimate central venous pressures one has to be mindful of technical limitations that can be observed for instance in patients with recent peripheral vein punctures for venous cannula placement or blood collection; this will very likely lead to vessel injury or spasm clouding the assessment of venous collapse and thus the evaluation of the arm with intact veins is preferred [49]–[52].

The use of non-invasive and, or invasive CVP measurements remain commonly utilized methods to estimate total intravascular volume. Care should be observed however in relying on single-point measurements since such a hemodynamic parameter can fluctuate over time based on variable clinical settings and may not necessary correlate with fluctuations in intravascular volume; serial evaluations of CVP along with integration with other cardiopulmonary physical exam findings are likely to yield more accurate estimates. CVP evaluation can also fail to correlate with intravascular volume in cases of elevated right-sided venous pressures due to specific pathologies such as decreased ventricular compliance, pulmonary artery hypertension, pulmonic or tricuspid valvular stenosis, and venous obstructions amongst others that do not necessarily specify an increased intravascular volume as opposed to pressure [53], [54].

There are other non-invasive techniques used to estimate central venous pressure that were not evaluated in this study. These include the use of controlled compression ultrasound evaluation of the great saphenous or peripheral arm veins [55]–[57]. Uthoff and colleagues prospectively studied the utility of forearm vein collapse evaluation in critically ill patients with an already indwelling central venous catheter measuring CVPs [57]. The proportion of identical CVP estimates by non-invasive ultrasound evaluation of forearm venous compression as compared to invasive measurements was modest at 61.4% [57]. Although such techniques have been reported in the literature, they are not commonly applied clinically at the bedside and require validation relative to other non-invasive techniques. They do nonetheless have merit and can be considered as adjunct tools when the evaluation of central venous pressures remains unclear.

## Conclusions

Noninvasive bedside evaluation of central venous pressure can be achieved by assessing the JVP, PVC techniques, and ultrasound visualization of the IVC. JVP evaluation is the most sensitive physical examination technique and its application improves with clinical experience. The PVC techniques along with the newly described Anthem sign may be of value for the early learner with limited experience with JVP assessments and in the evaluation of obese patients in whom JVP evaluation is difficult. Mini Echo estimates of CVP are comparable to physical examination assessments by trained clinicians and require less instruction. The use of Mini Echo in medical training should be further evaluated and encouraged.
